# Modified risk-stratified sequential treatment (subcutaneous rituximab with or without chemotherapy) in B-cell Post-transplant lymphoproliferative disorder (PTLD) after Solid organ transplantation (SOT): the prospective multicentre phase II PTLD-2 trial

**DOI:** 10.1038/s41375-022-01667-1

**Published:** 2022-08-16

**Authors:** Heiner Zimmermann, Christian Koenecke, Martin H. Dreyling, Christiane Pott, Ulrich Dührsen, Dennis Hahn, Norbert Meidenbauer, Ingeborg A. Hauser, Mathias J. Rummel, Dominik Wolf, Michael Heuser, Christian Schmidt, Peter Schlattmann, Matthias Ritgen, Reiner Siebert, Ilske Oschlies, Ioannis Anagnostopoulos, Ralf U. Trappe

**Affiliations:** 1 Department of Hematology and Oncology, DIAKO Ev. Diakonie-Krankenhaus, Bremen, Germany; 2 Pius-Hospital, University Medicine Oldenburg, Department of Hematology and Oncology, Oldenburg, Germany; 3 Department of Hematology, Hemostasis, Oncology and Stem Cell Transplantation, Hannover Medical School, Hannover, Germany; 4 Department of Internal Medicine III, LMU Klinikum, Munic, Germany; 5 Department of Internal Medicine II: Hematology and Oncology, University Medical Center Schleswig-Holstein, Campus Kiel, Kiel, Germany; 6 Department of Hematology, Essen University Hospital, University of Duisburg-Essen, Essen, Germany; 7 Department of Hematology, Oncology and Palliative Care, Katharinenhospital, Stuttgart, Germany; 8 Department of Medicine 5, University of Erlangen-Nuremberg, Erlangen, Germany; 9 Department of Nephrology, UKF, Goethe University Frankfurt, Frankfurt/Main, Germany; 10 Department of Hematology, Clinic for Haematology and Medical Oncology, Justus Liebig University Hospital, Gießen, Germany; 11 Internal Medicine 3, Hematology, Oncology, Immunooncology and Rheumatology, University Hospital Bonn, Bonn, Germany; 12 University Clinic V, Department of Hematology and Oncology, and Comprehensive Cancer Center Innsbruck (CCCI), Medical University Innsbruck (MUI), Innsbruck, Austria; 13 Institute of Medical Statistics, Computer and Data Sciences, Jena University Hospital, Jena, Germany; 14 Institute of Human Genetics, Ulm University and Ulm University Medical Center, Ulm, Germany; 15 Department of Hematopathology, University Medical Center Schleswig-Holstein, Campus Kiel, Kiel, Germany; 16 Institute of Pathology, University of Würzburg, Würzburg, Germany

**Keywords:** B-cell lymphoma, Combination drug therapy, Phase II trials

## Abstract

The prospective multicentre Phase II PTLD-2 trial (NCT02042391) tested modified risk-stratification in adult SOT recipients with CD20-positive PTLD based on principles established in the PTLD-1 trials: sequential treatment and risk-stratification. After rituximab monotherapy induction, patients in complete remission as well as those in partial remission with IPI < 3 at diagnosis (low-risk) continued with rituximab monotherapy and thus chemotherapy free. Most others (high-risk) received R-CHOP-21. Thoracic SOT recipients who progressed (very-high-risk) received alternating R-CHOP-21 and modified R-DHAOx. The primary endpoint was event-free survival (EFS) in the low-risk group. The PTLD-1 trials provided historical controls. Rituximab was applied subcutaneously. Of 60 patients enrolled, 21 were low-risk, 28 high-risk and 9 very-high-risk. Overall response was 45/48 (94%, 95% CI 83–98). 2-year Kaplan–Meier estimates of time to progression and overall survival were 78% (95% CI 65–90) and 68% (95% CI 55–80) – similar to the PTLD-1 trials. Treatment-related mortality was 4/59 (7%, 95% CI 2–17). In the low-risk group, 2-year EFS was 66% (95% CI 45–86) versus 52% in the historical comparator that received CHOP (*p* = 0.432). 2-year OS in the low-risk group was 100%. Results with R-CHOP-21 in high-risk patients confirmed previous results. Immunochemotherapy intensification in very-high-risk patients was disappointing.

## Introduction

Post-transplant lymphoproliferative disorders (PTLD) are complications of immunosuppression after solid organ transplantation (SOT). Their epidemiology, pathogenesis, classification, presentation and treatment have previously been reviewed in depth [1–5].

Two phase II trials conducted by the German PTLD study group and European PTLD Network established an evidence-based treatment protocol for CD20-positive B-cell PTLD in adults [6]. They demonstrated improved median overall survival (OS) compared to preceding smaller rituximab monotherapy trials [7–9] and lower treatment-related mortality (TRM) compared to prior retrospective case series of first-line chemotherapy [10–12]. The PTLD-1 sequential treatment (ST) trial (*n* = 70) demonstrated the safety and efficacy of 4 cycles of weekly rituximab followed by four cycles of CHOP-21 chemotherapy (cyclophosphamide, doxorubicin, vincristine, and prednisone, every 21 days) and established response to rituximab induction as a prognostic factor [13]. The subsequent trial of risk-stratified sequential treatment (PTLD-1 RSST, *n* = 152) established rituximab monotherapy consolidation for patients in a complete remission (CR) after rituximab induction and R-CHOP-21 (rituximab and CHOP-21) for all non-CR patients [14]. The question raised by this result was whether rituximab monotherapy consolidation might be sufficient treatment for additional PTLD patients. Further analyses of the initial PTLD-1 ST trial identified the prognostic value of international prognostic index (IPI) risk factors (≥3 vs <3) and thoracic SOT in addition to response to rituximab [15].

The PTLD-2 trial therefore tested modified risk-stratification based on these three clinical risk factors. The key hypothesis was that rituximab monotherapy consolidation in an expanded low-risk group would be superior to CHOP consolidation in comparable patients of the PTLD-1 ST trial by demonstrating an improved event-free survival (EFS) at 2 years, based on a lower rate of grade 3/4 infections and similar efficacy. The low-risk group receiving rituximab monotherapy consolidation was expanded compared to the RSST protocol by adding patients who had reached a partial remission after rituximab induction and had <3 IPI risk factors at diagnosis.

Thoracic organ transplant was a prognostic factor for shorter TTP in rituximab non-responders in the PTLD-1 ST trial [15]. Thus, we hypothesized that patients with both thoracic SOT and disease progression under rituximab induction might benefit from intensified immunochemotherapy. Six cycles of alternating R-CHOP and modified R-DHAOx every three weeks were chosen for this very-high-risk group, the latter based on efficacy and low renal toxicity. The R-DHAOx regime was modified due to the high risk of infectious complications in SOT recipients: the cytarabine dose was reduced to 50% and dexamethasone was reduced to one dose instead of four.

Finally, rituximab at a dose of 1400 mg SC was chosen based on its non-inferior pharmacokinetic profile compared to 375 mg/m^2^ intravenously (IV) in immunocompetent patients with follicular lymphoma and reports of rituximab IV serum concentrations during immunochemotherapy correlating with patient sex and clinical response [16, 17].

## Methods

### Study design and patients

This prospective, multicentre, open-label phase II trial enrolled treatment-naïve adult SOT recipients diagnosed with CD20-positive PTLD at 15 centres in Germany from February 3rd 2015 until July 13th 2020. Additional inclusion criteria were an insufficient response to upfront immunosuppression reduction (with or without antiviral therapy), measurable disease > 2 cm in diameter (and/or bone marrow involvement) and an Eastern Cooperative Oncology Group (ECOG) performance status ≤2. Main exclusion criteria were central nervous system involvement, pregnancy or nursing, and concomitant disease that precluded the administration of study therapy, in particular severe uncontrolled heart disease, HIV infection and other severe, active infections. Diagnostic tissue samples were collected and stored centrally, reviewed by two expert haematopathologists and classified according to the WHO classification [2]. Epstein–Barr virus association was confirmed by in-situ hybridization for EBER-transcripts. Fluorescence in-situ hybridization (FISH) was performed to identify MYC-, BCL2-, and BCL6- translocations as well as 11q aberrations if sufficient material was available. For details of staging, response assessments and follow-up examinations see the Supplementary Appendix. The responsible local ethics committees approved the trial and all patients gave written informed consent according to the Declaration of Helsinki. The trial is registered with clinicaltrials.gov (NCT02042391).

### Treatment plan

**Fig. 1:**
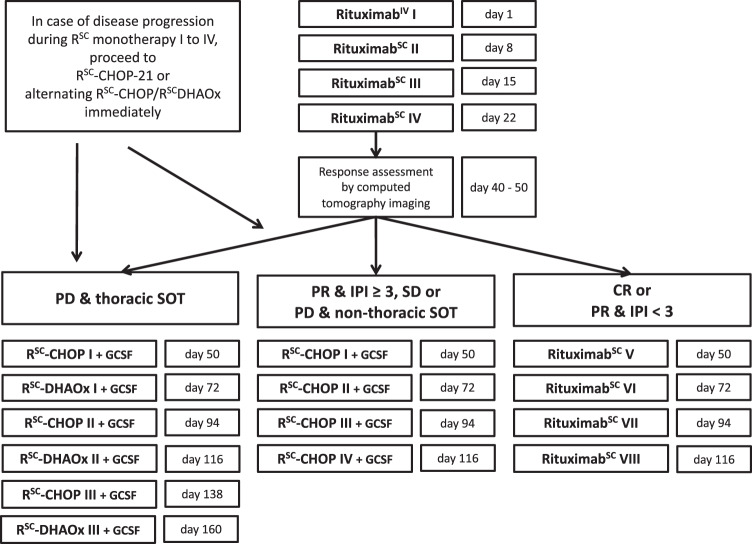
Modified risk-stratified sequential treatment schedule. Rituximab^IV^ denotes rituximab 375 mg/m^2^ IV and Rituximab^SC^ denotes rituximab 1400 mg SC. PD denotes progressive disease, SD stable disease, PR partial remission and CR complete remission. IPI ≥ 3/<3 denotes three or more/less than three international prognostic index risk factors at diagnosis (age > 60 years, Ann Arbor stage ≥III, ECOG performance status ≥2, elevated LDH, and more than one extranodal disease manifestation). R^SC^-CHOP-21: rituximab 1400 mg SC day (d) 1, cyclophosphamide 750 mg/m^2^ IV d1, doxorubicin 50 mg/m^2^ IV d1, vincristine 1.4 mg/m^2^ (max. 2 mg, 1 mg if over 70 years) IV d1, and prednisone 50 mg/m^2^ PO d1-5, every 21 days). R^SC^-DHAOx is modified R-DHAOx: rituximab 1400 mg SC day d1, oxaliplatin 130 mg/m^2^ IV d1, cytarabine 2 × 1000 mg/m^2^ IV d2, dexamethasone 40 mg PO d1. In case of progressive disease from d1 to d50, patients proceeded to R-CHOP-21 immediately. Thoracic SOT recipients are all patients after heart, lung or any other solid organ combined with a heart or a lung transplant. *Dose reduction of 50% in cycle 1 was recommended for patients with ECOG > 2.

Treatment (Fig. 1) consisted of rituximab (1400 mg SC; first application 375 mg/m^2^ IV) on days 1, 8, 15 and 22, followed by interim staging (day 40–50). Response to treatment at interim and final staging was determined according to PTLD-adapted response criteria for malignant lymphomas based on computed tomography imaging [18]. Patients in CR as well as those in partial remission (PR) with <3 international prognostic index (IPI) risk factors (age > 60 years, Ann Arbor stage ≥ III, ECOG performance status ≥2, elevated serum lactate dehydrogenase activity [LDH], and more than one extranodal disease manifestation) at diagnosis (low-risk group) continued with four three-weekly courses of rituximab [19]. Most other patients (high-risk group) received four cycles of R^SC^-CHOP-21 (for doses see Supplementary Appendix). Thoracic SOT recipients who progressed under rituximab (very-high-risk group) received six cycles of alternating R^SC^-CHOP and modified R^SC^-DHAOx in three-week intervals. In case of clinical signs of disease progression prior to interim staging, restaging was performed prematurely and treatment in the high-risk or very-high-risk groups started immediately if disease progression was confirmed. Similar to the PTLD-1 trials, *pneumocystis jirovecii* chemoprophylaxis and treatment with granulocyte-colony stimulating factor after chemotherapy were obligatory. The final response assessment was performed one month after the last cycle of therapy. Patients completed follow-up examinations every three months for two years, and annually thereafter. Follow-up data was evaluated up to July 2021. Median follow-up for the whole trial cohort as well as the low- and high-risk groups was 2.8 years (4.9 years for the very-high-risk group).

### Statistical analysis

Analysis was by intention to treat (ITT) unless otherwise specified. The per-protocol (PP) population included all patients treated according to protocol with a minimum number of two treatment cycles and sufficient information to determine remission status. For the calculation of the overall response to full treatment, subjects non-evaluable for response (i.e. subjects suffering TRM before final staging without evidence for PD) were not included in the denominator. The primary endpoint was EFS in the low-risk group. Events were infections grade III/IV from day 50 to day 143, treatment discontinuation for any reason, disease progression and death. The pre-specified comparator group included 25 patients in CR or PR with <3 initial IPI risk factors after rituximab IV induction who were treated with CHOP consolidation in the PTLD-1 ST trial [13]. Secondary endpoints included overall response rate (ORR), OS, time to progression (TTP), progression-free survival (PFS), response duration (RD) and TRM overall and by risk group. OS was defined from start of treatment to death from any cause (all patients), TTP from start of treatment to disease progression (all patients), PFS from start of treatment to disease progression or death (all patients) and RD from complete or partial response at final staging to disease progression (responding patients only). TRM was assessed by the treating physician. Adverse events were documented according to the Common Terminology Criteria, Version 4.0 (CTCAE; NCI CTEP, Bethesda, MD, USA). Pre-specified subgroup analyses were performed according to EBV-association and sex. Results from the PTLD-1 trials (ST and RSST) and relevant subgroups based on rituximab response, IPI and transplanted organ were used for pre-specified historical comparisons of efficacy, survival and toxicity, including an inter-trial comparison to detect sex-specific differences in the response to rituximab SC compared to rituximab IV [13, 14]. For sample size calculation and details on statistical tests see Supplementary Appendix.

## Results

### Patients

Baseline characteristics of the 60 patients enrolled are summarised in Table 1. Median time of follow-up was 2.8 years.

**Table 1:** Baseline characteristics of the 60 patients enrolled (ITT population).

Age/years: median (range)	53.8 (18–79)
≥60 years	18/60 (30%)
Male	44/60 (73%)
Transplant type	
Kidney	29/60 (48%)
Lung	14/60 (23%)
Liver	9/60 (15%)
Heart	4/60 (7%)
Kidney + pancreas	2/60 (3%)
Liver + kidney	1/60 (2%)
Liver + lung	1/60 (2%)
Time from transplantation to PTLD/years: median (range)	8.6 (0.3–31.2)
<1 year	13/60 (22%)
≥1 year	47/60 (78%)
Histology	
Polymorphic	2/60 (3%)
Monomorphic	58/60 (97%)
Burkitt lymphoma	2/60 (3%)
Burkitt-like lymphoma with 11q aberration	2/60 (3%)
High-grade B-cell lymphoma with *MYC* rearrangement	2/60 (3%)
DLBCL	45/60 (75%)
Marginal Zone Lymphoma	3/60 (5%)
Other monomorphic PTLD, CD20 positive	3/60 (5%)
Plasmacytoma, CD20-negative^a^	1/60 (2%)
EBV-association	
EBV-associated	23/60 (38%)
Non-EBV-associated	37/60 (62%)
Ann Arbor stage	
I	7/60 (12%)^b^
II	9/60 (15%)
III	6/60 (10%)
IV	38/60 (63%)
LDH	
Elevated	34/60 (57%)
Nodal disease	46/60 (76%)
Extranodal disease	56/60 (93%)
Gastrointestinal	28/60 (47%)
Liver	16/60 (27%)
Lung	11/60 (18%)
Kidney	3/60 (5%)
Bone marrow	6/60 (10%)
Graft	10/60 (17%)^c^
International Prognostic Index (IPI)	
<3	37/60 (62%)
≥3	23/60 (38%)
ECOG performance status	
0	22/60 (37%)
1	25/60 (42%)
2	13/60 (22%)

*DLBCL*: Diffuse large B-cell lymphoma, *EBV* Epstein–Barr Virus, *ECOG* Eastern Cooperative Oncology Group

^a^Diagnosis changed upon pathology review.

^b^All seven patients in stage IE.

^c^7/10 were lung transplant recipients.

### Treatment

**Fig. 2:**
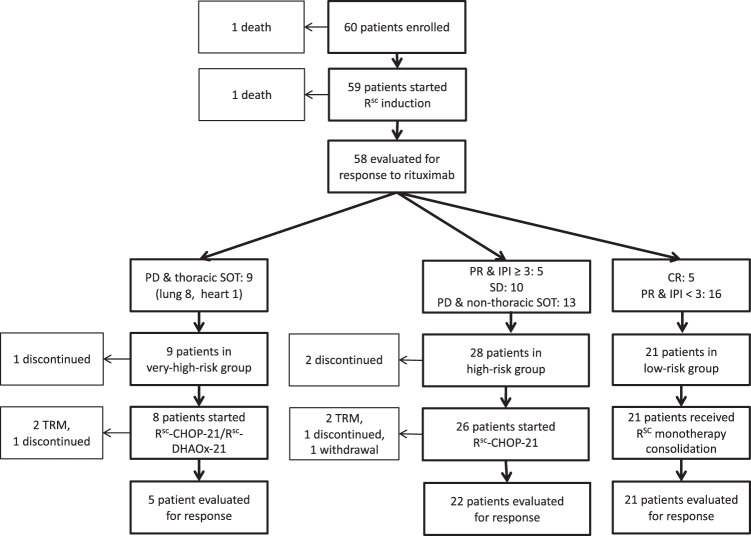
CONSORT diagram illustrating numbers of patients enrolled, treated and evaluated for response in the PTLD-2 trial. R^SC^ denotes rituximab 1400 mg SC. PD denotes progressive disease, SD stable disease, PR partial remission and CR complete remission. IPI refers to the number of international prognostic factor risk factors. These as well as the R^SC^-CHOP and R^SC^-DHAOx regimes are detailed in the Methods section. TRM denotes treatment-related mortality.

One patient died before the start of treatment, another during, but unrelated to rituximab induction (Fig. 2). 58 patients could be evaluated for response to rituximab induction, 48 of which had received all four scheduled applications. The ten patients that did not receive the four scheduled applications all had disease progression during treatment: three after the first application, two after two doses and four after three doses. In summary, 26/58 patients (45%, 95% CI 33–58) responded to rituximab monotherapy induction and 5/58 (9%, 95% CI 3–19) achieved a CR.

21 patients – five in CR and sixteen in PR with baseline IPI < 3 – were allocated to, received, and were evaluated after rituximab monotherapy consolidation in the low-risk group.

28 patients were allocated to the high-risk group due to PR with IPI ≥ 3 (five patients), stable disease (10 patients) or progressive disease (13 non-thoracic transplant recipients). 26/28 went on to receive treatment with R-CHOP-21 as two lung transplant recipients did not start treatment due to deteriorating transplant function and tuberculosis, respectively. Four patients discontinued treatment: One withdrew from trial treatment after one cycle and three patients stopped study treatment due to infections (fatal in two cases: liver abscesses and febrile neutropenia complicated by stroke, respectively). 22/28 patients could be evaluated at final staging.

Nine thoracic transplant recipients were allocated to the very-high-risk group due to disease progression at interim staging. Eight started treatment, as one lung transplant recipient declined further treatment in favour of best supportive care. Three patients discontinued treatment after the first cycle of R-CHOP due to infections (two cases of fatal neutropenic sepsis, one case of CMV-encephalitis). 5/9 patients could be evaluated at final staging.

In a comparison of baseline characteristics in the different risk groups (Table 2), we noted differences not only in baseline characteristics used for stratification (transplanted organ, IPI risk factors), but also in the rate of EBV-association and of early PTLD. None of the six patients with either Burkitt-, Burkitt-like- or high-grade BCL with MYC-rearrangement-type PTLD were treated in the low-risk-group. 8/15 lung transplant recipients were stratified to the very-high-risk group.

**Table 2:** Comparison of baseline patient characteristics in the low-risk (patients in CR after rituximab induction or PR with baseline IPI < 3), very-high-risk (thoracic transplant recipients with progressive disease after rituximab induction) and high-risk (all others) cohorts. 58 patients with restaging results after rituximab induction are included.

	Low-risk (*n* = 21)	High-risk (*n* = 28)	Very-high-risk (*n* = 9)	*p*
Age/years: median (range)	43.3 (18–79)	57.6 (18–72)	53.7 (24–62)	0.164
≥ 60 years	5/21 (24%)	11/28 (39%)	1/9 (11%)	0.213
Male	15/21 (71%)	22/28 (79%)	5/9 (56%)	0.402
Transplant type				
Kidney	13/21 (62%)	15/28 (54%)	0/9	<0.001
Lung	3/21 (14%)	3/28 (11%)	8/9 (89%)	
Liver	1/21 (5%)	8/28 (29%)	0/9	
Heart	1/21 (5%)	2/28 (7%)	1/9 (11%)	
Kidney + pancreas	2/21 (10%)	0/28	0/9	
Liver + lung	1/21 (5%)	0/28	0/9	
Time from transplantation to PTLD/years: median (range)	9.1 (0.3–31.2)	9.3 (0.5–24.7)	1.0 (0.4–13.5)	0.327
<1 year	6/21 (29%)	2/28 (7%)	5/9 (56%)	0.007
Histology				
Polymorphic	2/21 (10%)	0/28	0/9	
Monomorphic	19/21 (90%)	28/28 (100%)	9/9 (100%)	
Burkitt lymphoma	0/21	2/28 (7%)	0/9	
Burkitt-like lymphoma with 11q aberration	0/21	2/28 (7%)	0/9	
High-grade B-cell lymphoma with *MYC* rearrangement	0/21	1/28 (4%)	1/9 (11%)	
DLBCL	16/21 (76%)	20/28 (71%)	8/9 (89%)	
Marginal Zone Lymphoma	2/21 (10%)	1/28 (4%)	0/9	
Other monomorphic PTLD, CD20 positive	1/21 (5%)	2/28 (7%)	0/9	
EBV-association				
EBV-associated	11/21 (52%)	6/28 (21%)	6/9 (67%)	0.018
Ann Arbor stage				
I	4/21 (19%)	3/28 (11%)	0/9	0.246
II	5/21 (24%)	3/28 (11%)	1/9 (11%)	
III	1/21 (5%)	5/28 (18%)	0/9	
IV	11/21 (52%)	17/28 (61%)	8/9 (89%)	
LDH				
Elevated	6/21 (29%)	17/28 (61%)	9/9 (100%)	0.001
Nodal disease	14/21 (67%)	23/28 (82%)	7/9 (78%)	0.451
Extranodal disease	21/21 (100%)	24/28 (86%)	9/9 (100%)	0.100
Gastrointestinal	8/21 (38%)	15/28 (54%)	5/9 (56%)	
Liver	3/21 (14%)	7/28 (25%)	5/9 (56%)	
Lung	4/21 (19%)	2/28 (7%)	5/9 (56%)	
Kidney	2/21 (10%)	0/28	1/9 (11%)	
Bone marrow	2/21 (10%)	2/28 (7%)	1/9 (11%)	
Graft	4/21 (19%)	2/28 (7%)	4/9 (44%)	
International Prognostic Index (IPI)				
≥3	2/21 (10%)	13/28 (46%)	7/9 (78%)	0.001
ECOG performance status				
0	9/21 (43%)	11/28 (39%)	2/9 (22%)	0.302
1	10/21 (48%)	10/28 (36%)	3/9 (33%)	
2	2/21 (10%)	7/28 (25%)	4/9 (44%)	

*DLBCL* diffuse large B-cell lymphoma, *EBV* Epstein–Barr Virus, *ECOG* Eastern Cooperative Oncology Group.

### Outcome and toxicity

**Fig. 3:**
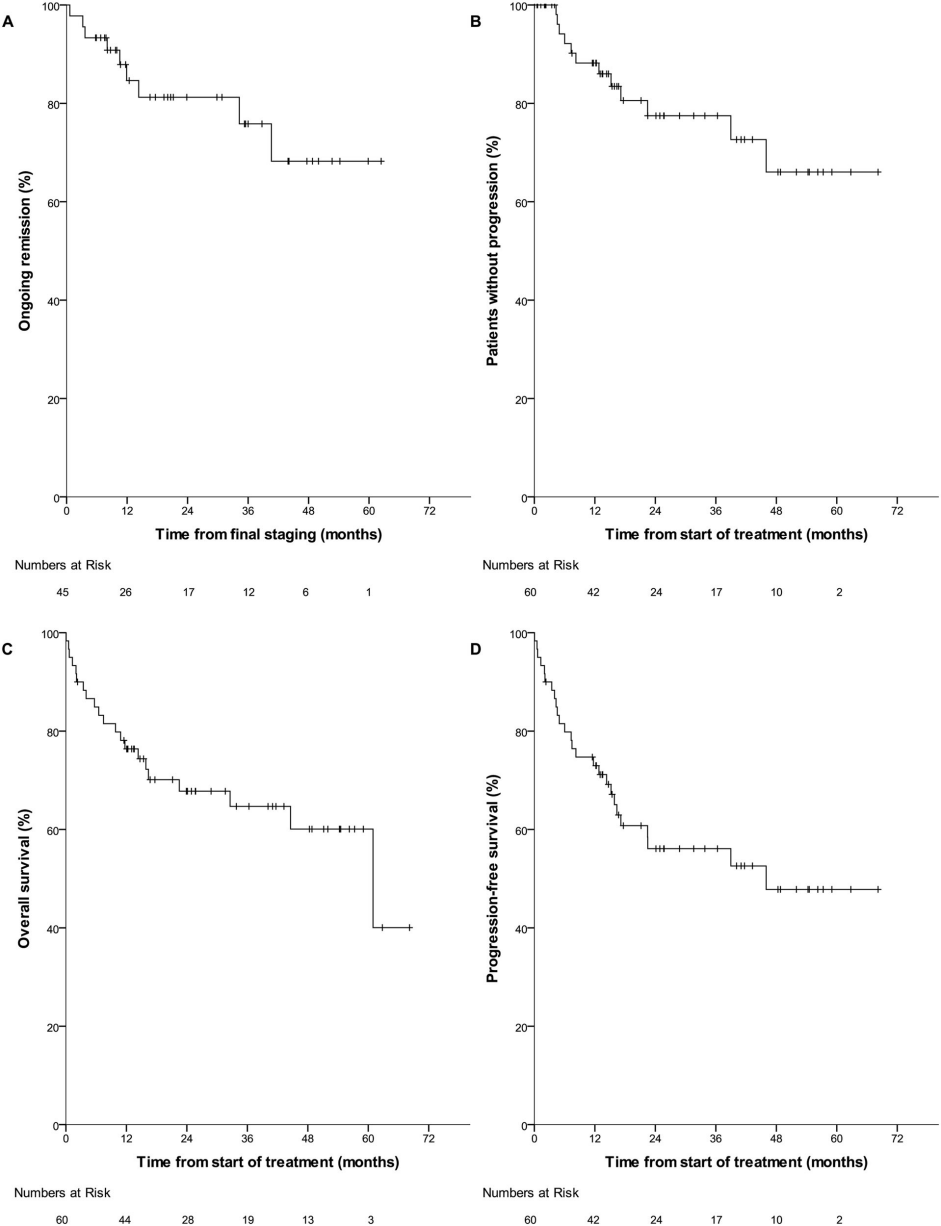
Time-to event outcomes in the intention-to treat population of the PTLD-2 trial (*n* = 60, except Fig. 3A). Median time of follow-up was 2.8 years. Numbers at risk are indicated at the bottom of each graph. **A** Response duration (patients in CR or PR, *n* = 45). **B** Time to progression. **C** Overall survival. **D** Progression-free survival.

The overall response rate at final staging was 94% (45/48, 95% CI 83–98; CR: 46% [22/48, 95% CI 33–60]). Median RD (Fig. 3A) was not reached. The 2-year Kaplan–Meier (KM) estimate of RD was 81% (95% CI 68–94). In the intention-to-treat population (60 patients), the 2-year KM estimate of TTP was 78% (95% CI 65–90, median not reached, Fig. 3B). The 2-year KM estimate for OS was 68% (95% CI 55–80, median 5.1 years, Fig. 3C). Median PFS (Fig. 3D) was 3.8 years with a 2-year KM estimate of 56% (95% CI 43–69). Toxicity of treatment was significant. Grade 3/4 leukopenia was reported in 22/59 (37%, 95% CI 26–50) patients at risk while grade 3/4 infections were reported in 25/59 (42%, 95% CI 31–55). Seven patients suffered more than one such infection (up to four). Most common episodes were pneumonia (nine), febrile neutropenia (eight), sepsis (six) and varicella zoster reactivation (three). There were two episodes each of influenza and fungal pneumonia as well as one case each of tuberculosis and CMV encephalitis, but no cases of *pneumocystis jirovecii* pneumonia or progressive multifocal leukoencephalopathy. Other frequent grade 3/4 adverse events included anaemia, thrombocytopenia, acute renal failure and gastrointestinal haemorrhage (see Table 3). Infusion-related reactions to rituximab IV occurred in 8/59 patients (14%, 95% CI 7–25; all grade 1 or 2) and local reactions to rituximab SC in 4/59 patients (7%, 95% CI 2–17; all grade 1). TRM affected 4/59 patients (7%, 95% CI 2–17), all after immunochemotherapy: Three patients died from infections, and one from stroke (following an infection after chemotherapy). A complete list of adverse events is provided in Supplementary Table 5.

**Table 3:** Adverse events grade 3 or 4 with a frequency >2% in the at-risk population of the PTLD-2 trial (*n* = 59).

Adverse event grade 3/4	Affected patients/patients at risk	Affected patients/patients at risk: low-risk group	Affected patients/patients at risk^a^: high-risk group	Affected patients/patients at risk^a^: very-high-risk group
Infection	25/59 (42%)	7/21 (33%)	12/26 (46%)	5/8 (63%)
Multiple episodes of infection	7/59 (12%)	0/21 (0%)	4/26 (15%)	3/8 (38%)
Leukopenia	22/59 (37%)	0/21 (0%)	16/26 (62%)	6/8 (75%)
Anemia	14/59 (24%)	0/21 (0%)	8/26 (31%)	6/8 (75%)
Thrombocytopenia	13/59 (22%)	0/21 (0%)	6/26 (23%)	7/8 (88%)
Acute renal failure	9/59 (15%)	1/21 (5%)	4/26 (15%)	4/8 (50%)
Gastrointestinal haemorrhage	4/59 (7%)	0/21 (0%)	3/26 (12%)	1/8 (13%)
Constipation	3/59 (5%)	0/21 (0%)	2/26 (8%)	1/8 (13%)
Diarrhoea	3/59 (5%)	0/21 (0%)	3/26 (12%)	0/8 (0%)
Tumour lysis syndrome	2/59 (3%)	0/21 (0%)	2/26 (8%)	0/8 (0%)
Multi-organ failure	2/59 (3%)	0/21 (0%)	0/26 (0%)	1/8 (13%)
Gastrointestinal perforation	2/59 (3%)	0/21 (0%)	1/26 (4%)	1/8 (13%)
Emesis	2/59 (3%)	1/21 (5%)	0/26 (0%)	1/8 (13%)

^a^Two patients in the high-risk group discontinued treatment before start of chemotherapy and one patient in the very-high-risk group discontinued treatment before start of chemotherapy. One of the 59 patients at risk was not allocated to a risk group.

We compared the outcomes of the PTLD-2 trial to the ST and RSST cohorts of the PTLD-1 trials (see Supplementary Appendix). 2-year KM estimates of DR, TTP, OS and PFS in the PTLD-1 ST and RSST cohorts were within the 95% CI reported for the PTLD-2 trial (Supplementary Fig. S1).

### Outcome and toxicity by treatment group

**Fig. 4:**
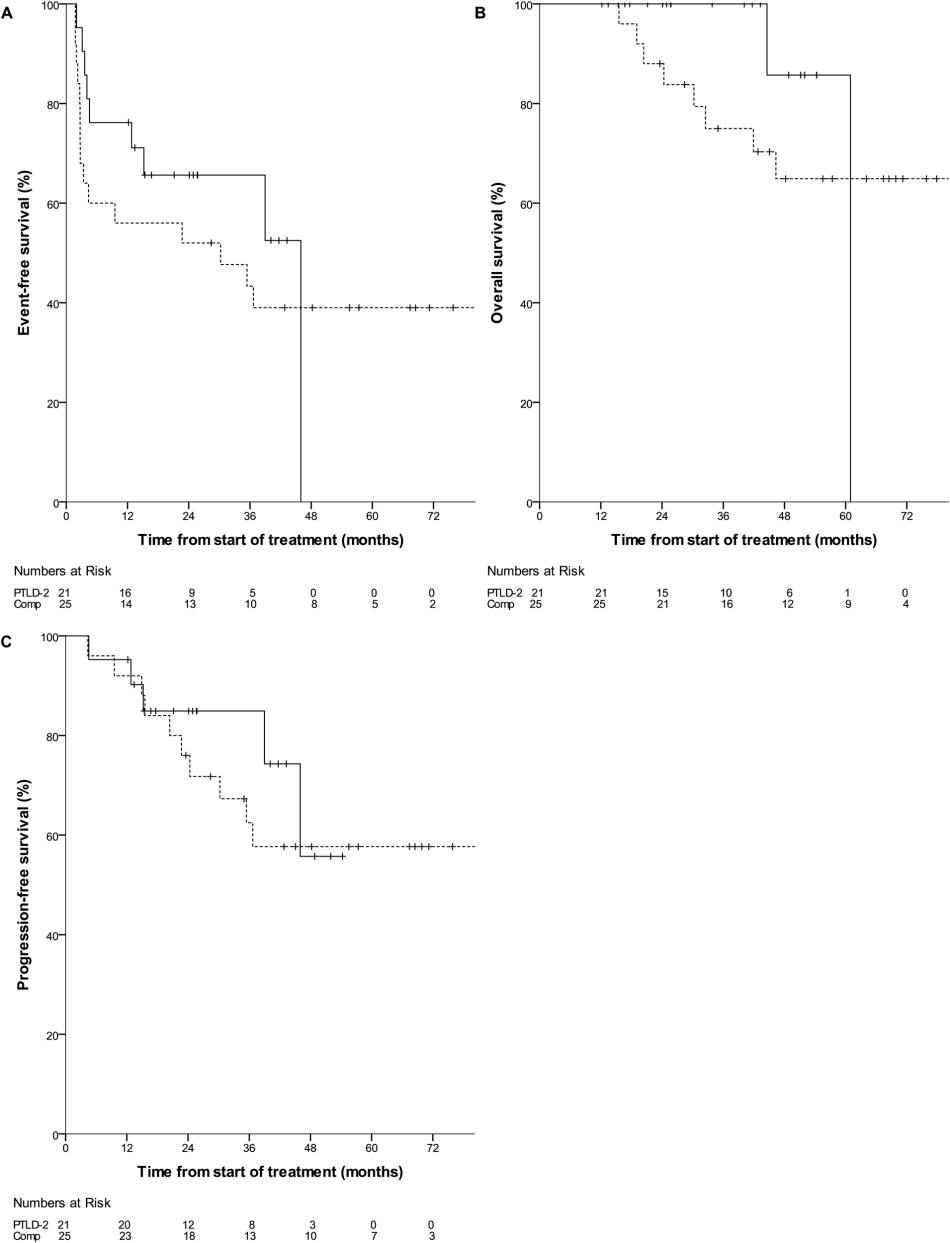
Event-free survival, overall survival and progression-free survival in the PTLD-2 low-risk group (*n* = 21, solid line). The comparator group (*n* = 25, broken line) are patients in CR (or PR with <3 initial IPI risk factors) after rituximab IV induction who were treated with CHOP consolidation in the PTLD-1 ST trial [13]. Events were infections grade 3/4 from day 50 to day 143, treatment discontinuation for any reason, disease progression and death. Numbers at risk for both populations (PTLD-2 and comparator) are indicated at the bottom of each graph. **A** Event-free survival (*p* = 0.432). The 2-year Kaplan–Meier EFS estimate in the low-risk group was 66% (95% CI 45–86); comparator group 52% (95% CI 32–72). **B** Overall survival (*p* = 0.324). The 2-year Kaplan–Meier OS estimate in the low risk group was 100%; comparator 88% (95% CI 75–100). **C** Progression-free survival (*p* = 0.597). The 2-year Kaplan–Meier PFS estimate in the low-risk group was 85% (95% CI 69–100); comparator group 76% (95% CI 59–93).

The overall response rate to rituximab monotherapy at interim staging was 100% in the low-risk group, 18% in the high-risk group and 0% in the very-high-risk group. Time-to event outcomes RD, TTP, OS and PFS for each risk group are plotted in Supplementary Figure S2. In the low-risk group, ORR at final staging was 95% (20/21 patients; CR 11/21, 52%). One patient suffered disease progression. Median RD was not reached; the 2-year KM estimate was 89% (95% CI 75–100). The primary endpoint of this trial, the 2-year EFS KM estimate in the low-risk group, was 66% (95% CI 45–86). This was 14% higher than in the pre-specified comparator group (52% [95% CI 32–72], Fig. 4A and Supplementary Appendix), but did not reach statistical significance (*p* = 0.432). EFS events included 4 grade 3/4 infections from day 50 to day 143 (19% vs. 32% in the comparator group). The 2- and 3-year KM estimates of both TTP and PFS were 85% (95% CI 69–100, median not reached in both cases, Fig. 4C). 2-year PFS in the comparator group was 76% (95% CI 59–93, *p* = 0.597, Fig. 4C). The 2- and 3-year KM estimate of OS was 100%; in the comparator group, 2-year OS was 88% (95% CI 75–100, *p* = 0.324, Fig. 4B). Toxicity was low; 7/21 (33%) patients suffered a single episode each of grade 3/4 infections (pneumonia in 4/7, none fatal) – three of these were outside the time interval defined for EFS. There was no TRM (0/21, 0%). Only three further adverse events grade 3/4 were reported.

In the high-risk group, ORR at final staging was 100% (22/22; CR 9/22 [41%]). Median RD was not reached; the 2- and 3-year KM estimate was 79% (95% CI 59–98). The 2- and 3-year KM estimate of TTP was 81% (95% CI 63–98, median not reached). The 2- and 3-year KM estimate of PFS was 54% (95% CI 34–75, median not reached). The 2- and 3-year KM estimate of OS was 59% (95% CI 39–79, median not reached). Chemotherapy in the high-risk group was associated with significant toxicity: 13/26 (50%) patients suffered grade 3/4 infections. Most frequent was febrile neutropenia. Haematotoxicity was common (leukopenia grade 3/4 in 16/26 [62%]), as was acute renal failure (4/26, 15%), gastrointestinal bleeding (3/26, 12%) and tumour lysis syndrome (2/26, 8%). TRM was 2/26 (8%).

In the very-high-risk group, 5/9 patients could be evaluated for response at final staging. 3/5 (60%) responded with two patients (40%) in CR. Median RD was 1.2 years. The 2-year KM estimate of TTP was 33% (95% CI 0–82). The 2-year KM estimate of PFS was 11% (95% CI 0–32, median 0.6 years) and median OS was 7.4 months (95% CI 2.2–12 months, 2-year KM estimate 30% [95% CI 0–62]. Toxicity in the very-high-risk group was substantial: 5/8 (63%) patients suffered grade 3/4 infections (two cases fatal, TRM 2/8 [25%]). Haematotoxicity was very common (leukopenia grade 3/4 in 6/8 [75%]), as was acute renal failure (4/8, 50%). Ten additional single cases of adverse events grade 3/4 were reported among these eight patients and included gastrointestinal bleeding, gastrointestinal perforation, multi-organ failure and cerebral bleeding. The highest rate of haematotoxicity, infections and mortality occurred after the first application of chemotherapy (R^SC^-CHOP) rather than subsequent cycles (either R^SC^-CHOP or R^SC^-DHAOx).

Response rates and time-to-event outcomes were confirmed by a per-protocol analysis (see Supplementary Appendix). The pre-specified subgroup analyses (EBV-association and sex) detected no significant differences in ORR, TTP or OS. There were no sex-specific differences in the response to rituximab SC compared to rituximab IV in a pre-specified inter-trial comparison with combined data from the PTLD-1 ST and PTLD-1 RSST trials (Supplementary Figs. S3 and S4).

### Prognostic factors

The three risk groups in this protocol had highly significantly different OS and PFS (*n* = 58, *p* < 0.001, Supplementary Fig. S2C, D), but not TTP (*n* = 58, *p* = 0.136, Supplementary Fig. S2B) or DR (*n* = 45, *p* = 0.905, Supplementary Fig. S2A). In a multivariate analysis (see Supplementary Appendix), only baseline IPI ≥ 3 remained an independent prognostic factor for both TTP and OS. Thoracic organ SOT was an independent prognostic factor for OS (Supplementary Table 4).

### Patients treated with rituximab monotherapy consolidation in the low-risk groups of PTLD-1 RSST and PTLD-2

**Fig. 5:**
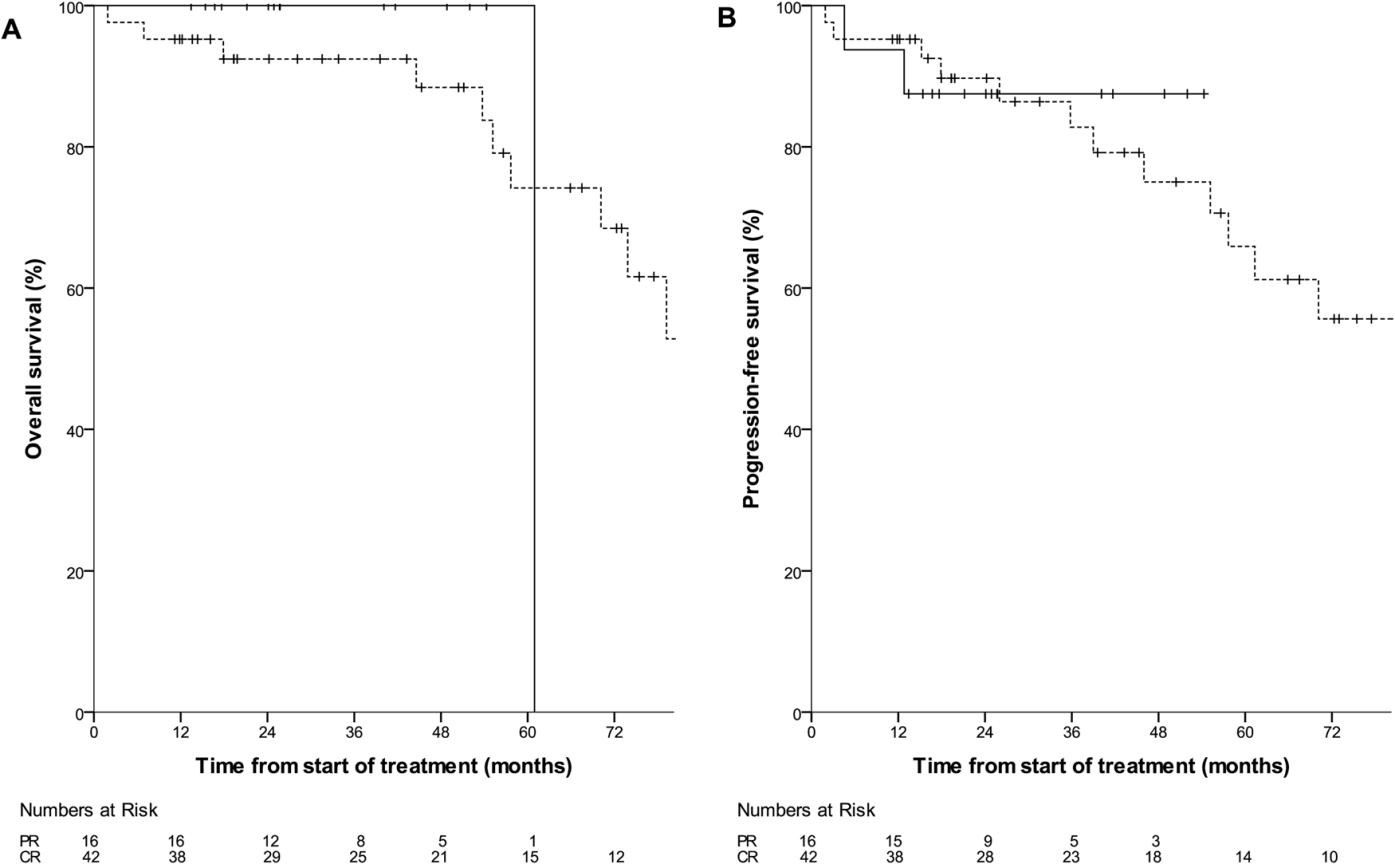
Overall and progression-free survival of 58 patients treated with rituximab monotherapy consolidation in the respective low-risk groups of the PTLD-1 RSST and PTLD-2 trials by complete or partial remission after rituximab induction. 42 patients in CR after rituximab induction in the PTLD-1 RSST (37 patients) and the PTLD-2 trials (5 patients) are denoted by a broken line. 16 patients in PR after rituximab induction with <3 IPI risk factors in the PTLD-2 trial are denoted by a solid line. Numbers at risk for both populations are indicated at the bottom of each graph. **A** Overall survival (*p* = 0.762). **B** Progression-free survival (*p* = 0.833).

To compare outcomes with rituximab monotherapy consolidation in patients in CR with those in PR after rituximab induction, we performed a pooled analysis of all patients treated with rituximab monotherapy consolidation in the respective low-risk groups of the PTLD-1 RSST and PTLD-2 trials (*n* = 58). 42 patients in CR after rituximab induction in the PTLD-1 RSST (37 patients) and the PTLD-2 trials (five patients) as well as 16 patients in PR after rituximab induction with <3 IPI risk factors treated in the PTLD-2 trial were included. Baseline characteristics of the total 58 patient cohort as well as the CR and PR subgroups are given in Supplementary Table 3. The only significant differences were found in the number of patients with ≥3 IPI risk factors (excluded from the PR group by definition) and in the presence of individual IPI risk factors (‘elevated serum LDH activity’, ‘extranodal disease’). There were no differences in overall survival (*p* = 0.762, 2-year-KM estimate 100% in the PR group and 92% [95% CI 84-100] in the CR group, Fig. 5A) or progression-free survival (*p* = 0.833, 2-year-KM estimate 88% [95% CI 71–100] in the PR group and 90% [95% CI 80–99] in the CR group Fig. 5B).

## Discussion

Rarity and disease heterogeneity are the reasons evidence for PTLD treatment is still based on phase II clinical trial data. The PTLD-1 trial set a new standard in CD20-positive B-cell PTLD by establishing sequential treatment with four courses of weekly rituximab followed by four cycles of CHOP-21 for patients unresponsive to initial immunosuppression reduction. With a median OS of 6.6 years and a clear plateau on the PFS curve it compared favourably to earlier rituximab monotherapy trials. The subsequent trial of risk-stratified sequential treatment (PTLD-1 RSST) successfully tested a response-adapted treatment strategy based on response to rituximab monotherapy induction. Exposure of patients to chemotherapy was limited and long-term outcomes maintained. It is considered a 1^st^-line standard in the treatment of PTLD [6].

This prospective, multicentre, phase II trial tested modified risk-stratified sequential treatment of CD20-positive PTLD in adults after SOT. We observed similar ORR, TTP, OS and PFS compared to the preceding PTLD-1 ST and RSST trials. 36% of patients were treated with rituximab monotherapy consolidation and thus chemotherapy-free in the low-risk group – 76% of them would have received R-CHOP consolidation using the preceding PTLD-1 RSST protocol. OS and PFS were significantly different in the three risk groups – however, DR as a measure of the quality of remissions was very similar.

2-year EFS in the low-risk group was numerically, but not statistically, superior to a pre-specified comparator group from the PTLD-1 ST trial treated with CHOP consolidation. Thus, the primary endpoint of the trial was not met and the original trial hypothesis (rituximab consolidation results in lower toxicity and similar efficacy) is rejected. Arguably, a major factor was the higher than anticipated rate of grade 3/4 infections in the low-risk group. Indeed, further examination of the data justifies the conclusion that rituximab monotherapy consolidation in an expanded low-risk group including patients with <3 IPI risk factors in PR after rituximab induction is a safe and feasible alternative to CHOP consolidation. These observations include the secondary endpoints OS (2-year estimate 100%) and PFS in this subgroup, the highly similar response duration in all three risk groups (*p* = 0.905) and a pooled analysis of all patients treated with rituximab monotherapy consolidation in the PTLD-1 RSST and PTLD-2 trials, which demonstrated similar PFS irrespective of CR or PR with <3 IPI risk factors after rituximab induction.

The PTLD-2 study used treatment stratification based on response to treatment at interim staging based on CT imaging rather than positron emission tomography (PET) to ensure that the protocol can be implemented worldwide and to maintain comparability with the earlier PTLD-1 trials. We have shown that end-of-treatment PET identifies patients at low risk of relapse and offers clinically relevant information in PTLD, particularly in patients in partial remission by CT imaging [20]. While some patients in partial remission after rituximab induction might hypothetically have been evaluated as complete remission by PET, it is unlikely that interim staging based on PET would have identified additional low-risk patients. Only 5/58 patients were considered high-risk based on a PR at interim staging and an IPI ≥ 3 at diagnosis (9%). Whether any of these patients would have been PET-negative is questionable.

The PTLD-2 trial extends the strategy of therapy de-escalation started in PTLD-1 RSST. Parallel to the introduction and expansion of rituximab monotherapy consolidation in patients considered low-risk based on response to rituximab, this risk factor has lost its independent positive prognostic value for TTP in multivariate analysis. In the PTLD-1 ST trial, the hazard ratio (HR) of overall response to rituximab induction for TTP was 0.213 (*p* = 0.008), increasing to 0.256 (*p* < 0.001) in the PTLD-1 RSST trial [14, 15]. In an analogous multivariate analysis in the PTLD-2 trial, overall response to rituximab was excluded from the multivariate model at step 1 with a HR of 0.867 (*p* = 0.811). We therefore suspect that there is little margin for further expansion of rituximab monotherapy consolidation beyond the low-risk group of the current trial.

Patient allocation to the very-high-risk group of the PTLD-2 protocol was based on two previously identified risk factors for poor outcome: progressive disease under rituximab induction and thoracic organ transplant [14, 15]. The results in this small group remain highly disappointing with six cycles of immunochemotherapy consolidation including oxaliplatin and cytarabine: high TRM (25%) and median PFS and OS estimates <1 year. With the caveat of a small sample, we conclude that intensified immunochemotherapy does not overcome the poor prognosis in this patient subgroup. Alternative treatment strategies are needed for this very-high-risk subgroup. For those with EBV-associated PTLD (6/9 in this trial), EBV-specific cytotoxic T-cells are a less toxic alternative based on a recent case series [21]. Efficacy is currently tested in an ongoing prospective trial (NCT03394365).

The independent prognostic value of ≥3 IPI risk factor for both OS and TTP in CD20-positive PTLD was confirmed in this trial, adding to the identical finding from the PTLD-1 ST and RSST cohorts as well as recent large real-world data sets of PTLD after SOT [14, 15, 22, 23]. Outcomes (ORR, TTP and OS) were virtually identical irrespective of EBV-association, in contrast to the considerable differences in genomic profiles [24, 25].

As in our preceding trials, the rate of grade 3/4 infections was high (42%). However, we observed no cases of pneumocystis jirovecii pneumonia under stringent antibiotic prophylaxis. TRM occurred exclusively in patients treated with immunochemotherapy with an overall rate of 7%, very similar to immunocompetent patients over 60 years treated with R-CHOP immunochemotherapy for DLBCL [26, 27].

This trial utilized rituximab in its formulation for subcutaneous injection. In a pre-specified inter-trial comparison, we did not detect significant differences in ORR between rituximab SC and IV, nor a sex-specific difference. This is in line with data reported for single agent rituximab in follicular lymphoma and in combination with CHOP in DLBCL in randomized trials of immunocompetent patients [28, 29]. However, CR rates after 4 weekly courses of rituximab monotherapy might be slightly lower with rituximab SC (see Supplementary Appendix).

Limitations of this trial included its small size and lack of international recruitment due to funding constraints. This may have resulted in the over-representation of lung transplant recipients (15/60, 25%), who have a poor prognosis [30], as well as the comparatively high proportion of Burkitt, Burkitt-like lymphoma with 11q aberration and high-grade B-cell lymphoma PTLD (6/60, 10%). However, the latter performed particularly well with 5/6 patients reaching long-term remission [31] (see Supplementary Appendix).

During the conduct of the PTLD-2 trial, two other prospective multicentre phase II trials evaluated ibrutinib and brentuximab vedotin, respectively, in the treatment of PTLD. Ibrutinib was added to risk-stratified sequential treatment in induction and consolidation (*n* = 39). It did not result in a sufficiently high CR rate to warrant further investigation, while the OS and PFS outcomes were very similar to those reported in the PTLD-1 RSST trial [32]. A small trial of brentuximab vedotin given concurrently with rituximab for patients with de novo immunosuppression-associated CD30-positive and/or EBV-positive lymphomas included 12 PTLD after solid organ transplantation [33]. Toxicity and efficacy for the PTLD subgroup were not analysed, and the evidence available does not challenge the concept of risk-stratified sequential treatment as first-line treatment of CD20-positive PTLD in adults after SOT.

## Supplementary information

Supplementary Appendix
