# Supplementary material for: Modified risk-stratified sequential treatment (subcutaneous rituximab with or without chemotherapy) in B-cell Post-transplant lymphoproliferative disorder (PTLD) after Solid organ transplantation (SOT): the prospective multicentre phase II PTLD-2 trial
Source: Leukemia. 2022 Aug 16;36(10):2468–78. doi: 10.1038/s41375-022-01667-1 (PMC9522585; doi:10.1038/s41375-022-01667-1)
Supplement: Supplementary file 1 — Supplementary Appendix [file 41375_2022_1667_MOESM1_ESM.docx]

**Supplementary Appendix to manuscript submitted by Zimmermann, H. et al:**

**Modified risk-stratified sequential treatment (subcutaneous rituximab with or without chemotherapy) in B-cell Post-transplant lymphoproliferative disorder (PTLD) after Solid organ transplantation (SOT): the prospective multicentre phase II PTLD-2 trial**

**Supplementary Methods**

Disease stage at enrolment was determined through a complete patient history, physical examination, laboratory investigations (including full blood count, lactate dehydrogenase [LDH] and renal and liver function tests), bone marrow biopsy, and contrast-enhanced computed tomography (CT) scans of the neck, chest and abdomen and pelvis as well as magnetic resonance tomography of the neurocranium. Blood samples for biomarker discovery were collected and stored centrally. Patients provided informed consent to storage and analysis of tissue and blood samples. Interim and final response assessment included a complete patient history, physical examination, laboratory investigations and CT scans of the neck, chest, abdomen and pelvis. At follow-up assessments, CT scans were substituted by plain chest radiographs and abdominal ultrasound, except at six, 12 and 24 months follow-up. Further investigations such as bone marrow biopsy, CT scans of the head or endoscopy were performed if clinically indicated to determine remission status.

R^SC^-CHOP dosing:
rituximab 1400 mg SC day (d) 1, cyclophosphamide 750 mg/m^2^ IV d1, doxorubicin 50 mg/m^2^ IV d1, vincristine 1.4 mg/m^2^ (max 2 mg, 1 mg if over 70 years) IV d1, and prednisone 50 mg/m^2^ PO d1-5, every 21 days)

R^SC^-DHAOx dosing:
rituximab 1400 mg SC day d1, oxaliplatin 130 mg/m^2^ IV d1, cytarabine 2x 1000 mg/m^2^ IV d2, dexamethasone 40 mg po d1

Sample size:
The sample size calculation was based on the assumption of a 2-year EFS of 82% versus 52% in the comparator group, based in turn on an assumed reduction of infections grade III/IV from 30% to 5%, a reduction of treatment discontinuation and a similar TTP. The trial protocol was amended in 2017 to account for a low initial rate of recruitment. The accrual period was extended to 72 months while the minimum follow-up time of 12 months was maintained. With a two-sided significance level set to p=0.050 and a power of 90% a total of 15 low-risk patients were needed to test the null hypothesis that the exponential parameter λ_0_=-ln(0.52)/24=0.027 versus the alternate λ_1_=-ln(0.82)/24=0.008. With a calculated rate of 38% of all patients stratified to the low-risk group and a safety margin of 33% this corresponds to a total of 60 patients needed to perform the trial.

Statistical calculations:
Confidence intervals and best point estimates for observed response rates were calculated using the adjusted Wald method (GraphPad Prism). Time-to-event outcomes were described using Kaplan-Meier statistics. Exploratory analyses were performed using two-sided stratified log-rank tests as well as the χ2 test for categorical variables and the independent-samples Kruskal-Wallis test for continuous variables. Multivariate analyses were performed using Cox-regression models (log-rank ratio test, backwards elimination). The two-sided significance level was set at 0.05 and statistical tests were performed using IBM SPSS 22.0.0.0 unless otherwise specified.

**Supplementary Results**

*Baseline characteristics in comparison to PTLD-1 sequential treatment (ST, Trappe et al., 2012) and PTLD-1 risk-stratified sequential treatment (RSST, Trappe et al., 2017)*

Baseline characteristics of all three trial cohorts are listed in Supplementary Table 1. We noted that the percentage of lung transplant recipients was higher in PTLD-2 (15/60, 25%) than in ST (6/70, 9%) or RSST (20/152, 13%). Conversely, the percentage of both heart and liver transplant recipients was lower. Furthermore, Burkitt-type PTLD, Burkitt-like PTLD with 11q aberration and high-grade B-cell lymphoma with *MYC* aberration taken together constituted 10% (6/60) of the trial ITT population (3% in ST, 4% in RSST). Finally, the rate of EBV-association was lowest in PTLD-2 (23/60, 38%) compared to ST (44%) and RSST (47%).

*Outcomes and toxicity in comparison to PTLD-1 sequential treatment (ST, Trappe et al., 2012) and PTLD-1 risk-stratified sequential treatment (RSST, Trappe et al., 2017)*

We compared the outcomes of the PTLD-2 trial cohort to the ST (Trappe et al., 2012) and RSST (Trappe et al., 2017) cohorts of the PTLD-1 trials.

The complete remission rate with rituximab induction in PTLD-2 was 9% (5/58, 95% CI 3-19). In comparison, complete remission rates with rituximab induction were 20% (14/70, 95% CI 12-31, p=0.141) in the PTLD-1 ST cohort and 25% (37/148, 95% CI 19-33, p=0.032) in the PTLD-1 RSST cohort.

The overall response rate at final staging in the PTLD-2 trial was 94% (45/48, 95% CI 83–98; CR: 46% [22/48, 95% CI 33–60]). In comparison, overall response rates were 53/59 (90%, 95% CI: 79-96%; CR: 40/59 (68%, 95% CI: 55-78%) in the PTLD-1 ST cohort and 88% (111/126, 95% CI 81–93; CR: 70% [88/126, 95% CI 61– 77]) in the PTLD-1 RSST cohort.

2-year KM estimates of DR, TTP, OS and PFS in the PTLD-1 ST and RSST cohorts were within the 95% CI reported for the PTLD-2 trial (see Supplementary Figure S1). The PTLD-2 2-year Kaplan-Meier (KM) RD estimate was 81% (95% CI 68–94; ST 80%, RSST 86%; p=0.494, Figure S1A). The PTLD-2 2-year KM estimate of TTP was 78% (95% CI 65–90; ST 71%; RSST 77%; p=0.551, Figure S1B). The PTLD-2 2-year KM estimate of OS was 68% (95% CI 55–80; ST 67%; RSST 72%; p=0.574, Figure S1C). The PTLD-2 2-year KM estimate of PFS was 56% (95% CI 43–69; ST 61%; RSST 67%; p=0.397, Figure S1D).

Grade 3 or 4 infections were reported in 25/59 patients (42%, 95% CI 31–55) in the PTLD-2 trial, compared to 26/64 patients (41%, 95% CI 29-53%) in PTLD-1 ST and 52/151 patients (34%, 95% CI 27–42) in the PTLD-1 RSST cohort.

Treatment-related mortality occurred in 4/59 pts (7%, 95% CI 2–17) in the PTLD-2 trial, compared to 11% (95% CI 5-21%) in the PTLD-1 ST and 8% (95% CI 5–14) in the PTLD-1 RSST cohort.

In summary overall response rates and time-to-event outcomes at 2 years as measures of efficacy, as well as the key toxicity parameters (rate of grade 3 or 4 infections and TRM) in the PTLD-1 ST and RSST cohorts were within the 95% CI reported for the PTLD-2 trial. The only notable difference was the rate of CR at final staging – however, there was no difference in RD.

*Baseline characteristics in the low-risk-group of the PTLD-2 trial compared to the pre-specified comparator group for EFS*

The pre-specified comparator group for EFS in the low-risk group were patients in CR (or PR with <3 initial IPI risk factors) after rituximab IV induction (n=25) who were treated with CHOP consolidation in the PTLD-1 sequential treatment trial (Trappe et al., 2012). The baseline characteristics of patients treated in the low-risk group of the PTLD-2 trial (n=21) are compared to those of the comparator group in Supplementary Table 2. Overall, this historical comparator group is well matched. Significant differences included a higher rate of patients in CR after rituximab induction in the comparator group (56% vs. 24%, p=0.027), a different distribution of transplanted organs (the comparator group had more heart and liver recipients and fewer kidney and lung recipients) as well a lower rate of extranodal disease in the comparator group (79% vs. 100%, p=0.027).

*Per-protocol analysis*

In the pre-specified per-protocol analysis, one patient who had violated inclusion criteria was excluded from all analyses due to a diagnosis of CD20-negative PTLD on pathology review. The very same patient died after consent and trial registration but before the start of protocol treatment due to extradural haemorrhage. There were no other patients who violated inclusion/exclusion criteria. Three additional patients were excluded from the per-protocol calculation of response rates at interim staging as they had received only one application of rituximab before treatment escalation to immunochemotherapy due to progressive disease (at least two applications were required per protocol). However, all three remained in the per-protocol analyses of time-to-event outcomes overall and for the risk groups.

Thus 59/60 patients were included in the per-protocol calculation of time-to-event outcomes (EFS, RD, TTP, OS, PFS). The protocol furthermore specified the censoring of patients at the time of protocol violation. This affected seven patients due to treatment discontinuation for any reason and two additional patients due to consolidating chemotherapy in partial remission (two cycles of rituximab, carboplatin and etoposide). Of note, patients who received second-line treatment due to refractory disease or relapse were not censored. No patients were excluded due to either treatment in the wrong risk-group, unjustified dose reductions or other critical protocol violations.

In the per-protocol analysis, the overall response rate to rituximab induction was 26/55 patients (47%, 95% CI 35–60). 5/55 patients (9%, 95% CI 4–20) achieved a CR at interim staging and 21/55 (38%, 95% CI 26–51) a PR. The response rate at final staging was identical to the intention-to-treat analysis: overall response 94% (45/48, 95% CI 83–98; CR: 46% [22/48, 95% CI 33– 60]).

Median RD was not reached; the 2-year Kaplan-Meier (KM) estimate was 83% (95% CI 70–95). The 2-year KM estimate of TTP was 77% (95% CI 64–91, median not reached). The 2-year KM estimate of OS was 74% (95% CI 61–87, median OS 5.1 years). Median PFS was not reached with a 2-year KM estimate of 60% (95% CI 46–75).

Per-protocol outcomes in the low-risk group (including low-risk EFS, the primary endpoint) were identical to the intention-to-treat population as no patients in the low-risk group were either excluded or censored. In the high-risk group, median RD was not reached; the 2- and 3-year KM estimate was 81% (95% CI 60–100). The 2- and 3-year KM estimate of TTP was 81% (95% CI 61–100, median not reached). The 2- and 3-year KM estimate of PFS was 54% (95% CI 30–79, median not reached). The 2- and 3-year KM estimate of OS was 61% (95% CI 37–84, median not reached). In the very-high-risk group, median RD was 1.2 years. The 2- and 3-year KM estimate of TTP was 30% (95% CI 0–80, median 1.9 years). The 2- and 3-year KM estimate of PFS was 15% (95% CI 0–42, median 0.6 years). Median OS was 1.2 years (95% CI 0–2.6 years). The 2- and 3-year KM estimates of OS were 40% (95% CI 0–62) and 20%, respectively.

In summary, the results of the per-protocol analysis closely matched and thus confirmed the results from the intention-to-treat population.

*Pre-specified subgroup analyses: EBV and sex*

Comparing EBV-positive and EBV-negative PTLD, we found no significant differences in response (ORR 17/19 [90%] vs. 28/29 [97%], p=0.322), TTP (p=0.952) or OS (p=0.867), see Supplementary Figure S3. Similarly, in a comparison by sex, there were no significant differences in ORR (females 12/13 [92%] vs. males 33/35 [94%], p=0.801), TTP (p=0.772) or OS (p=0.668), see Supplementary Figure S4. Moreover, ORR to rituximab induction was similar in male and female patients (20/42 [48%] vs. 6/16 [38%], p=0.489). In the pre-specified inter-trial comparison with combined data from the PTLD-1 ST and PTLD-1 RSST trials to detect gender-specific differences in the response to rituximab SC compared to rituximab IV, we did not detect significant differences in ORR to rituximab SC compared to IV: neither overall (26/58 [45%] vs. 122/218 [56%], p=0.131), nor among males (20/42 [48%] vs. 93/159 [59%], p=0.207) or females (6/16 [38%] vs. 29/59 [49%], p=0.407).

*Non-pre-specified subgroup analyses: Burkitt, Burkitt-like lymphoma with 11q aberration and high-grade B-cell lymphoma PTLD*

6 patients with Burkitt, Burkitt-like lymphoma with 11q aberration and high-grade B-cell lymphoma PTLD were included. None of these patients responded to rituximab monotherapy. 5/6 were allocated to the high-risk group and received R^SC^-CHOP-21. 1 patient was allocated to the very-high-risk group and received six cycles of alternating R^SC^-CHOP and modified R^SC^-DHAOx. There were 3 CRs and 2 PRs. 1 patient in the high-risk group stopped treatment prematurely after 3 cycles of R^SC^-CHOP-21 due to infection. 1 patient relapsed 3 months after completion of treatment and died from PTLD. 5/6 patients are alive without evidence of PTLD at 14, 32, 51, 59 and 63 months of follow up.

*Prognostic factors*

Individually, response to rituximab induction remained a significant predictor of OS (n=58, p=0.035) but not of PFS, TTP or DR. Baseline IPI ≥3 was associated with significantly poorer OS and PFS (n=60, p<0.001, Supplementary Figure 5B), TTP (p=0.016, Supplementary Figure 5A) and DR (p=0.017). Finally, thoracic SOT recipients had significantly poorer OS (p=0.006) and PFS (p=0.038), but not TTP or DR.

*Multivariate analysis of prognostic factors*

We performed a backward-step log-rank multivariate Cox regression analysis including the factors thoracic organ transplant, overall response to rituximab and IPI ≥3 in the PTLD-2 trial cohort. This demonstrated that only baseline IPI ≥ 3 was a significant independent prognostic factor for both OS and TTP in the 58/60 patients with available rituximab response data (Supplementary Table 4). Thoracic organ SOT was an independent prognostic factor for OS.

*References quoted in the supplementary appendix:*

Trappe, R., Oertel, S., Leblond, V., Mollee, P., Sender, M., Reinke, P., Neuhaus, R., Lehmkuhl, H., Horst, H.A., Salles, G., et al. (2012). Sequential treatment with rituximab followed by CHOP chemotherapy in adult B-cell post-transplant lymphoproliferative disorder (PTLD): the prospective international multicentre phase 2 PTLD-1 trial. The Lancet Oncology *13*, 196–206.

Trappe, R.U., Dierickx, D., Zimmermann, H., Morschhauser, F., Mollee, P., Zaucha, J.M., Dreyling, M.H., Dührsen, U., Reinke, P., Verhoef, G., et al. (2017). Response to Rituximab Induction Is a Predictive Marker in B-Cell Post-Transplant Lymphoproliferative Disorder and Allows Successful Stratification Into Rituximab or R-CHOP Consolidation in an International, Prospective, Multicenter Phase II Trial. J. Clin. Oncol. *35*, 536–543.

**Supplementary Table 1:** Baseline characteristics: Comparison of patients treated 2015-2020 in the PTLD-2 trial and patients treated in the PTLD-1 trials 2002-2008 with sequential treatment (Trappe et al., 2012) and 2006-2014 with risk-stratified sequential treatment (Trappe et al., 2017).

|  | **PTLD-1, sequential treatment (n=70)** (Trappe et al., 2012) | **PTLD-1, risk-stratified sequential treatment (n=152)** (Trappe et al., 2017) | **PTLD-2 (n=60)** |
| --- | --- | --- | --- |
| **Age/years: median (range)** | 53.3 (16–74) | 56.4 (18–82) | 53.8 (18–79) |
| ≥ 60 years | 21/70 (30%) | 60/152 (40%) | 18/60 (30%) |
| **Male** | 47/70 (67%) | 115/152 (76%) | 44/60 (73%) |
| **Transplant type** | | | |
| Kidney | 29/70 (41%) | 69/152 (45%) | 29/60 (48%) |
| Liver | 16/70 (23%) | 40/152 (26%) | 9/60 (15%) |
| Heart | 14/70 (20%) | 15/152 (10%) | 4/60 (7%) |
| Lung | 4/70 (6%) | 18/152 (12%) | 14/60 (23%) |
| Heart + kidney | 0 | 5/152 (3%) | 0 |
| Kidney + pancreas | 4/70 (6%) | 3/152 (2%) | 2/60 (3%) |
| Heart + lung | 2/70 (3%) | 2/152 (1%) | 0 |
| Liver + kidney | 0 | 0 | 1/60 (2%) |
| Liver + lung | 0 | 0 | 1/60 (2%) |
| Bone marrow | 1/70 (1%) | 0 | 0 |
| **Time from transplantation to PTLD/years: median (range)** | 5.5 (0.1–25.3) | 9.0 (0.2–27.9) | 8.6 (0.3-31.2) |
| < 1 year | 17/70 (24%) | 32/152 (21%) | 13/60 (22%) |
| ≥ 1 year | 53/70 (76%) | 120/152 (79%) | 47/60 (78%) |
| **Histology** | | | |
| Early lesion | 0 | 2/152 (1%) | 0 |
| Polymorphic | 3/70 (4%) | 20/152 (15%) | 2/60 (3%) |
| Monomorphic | 67/70 (96%) | 129/152 (85%) | 58/60 (97%) |
| Burkitt and Burkitt-like with 11q aberration | 2/70 (3%) | 6/152 (4%) | 4/60 (7%) |
| High-grade B-cell lymphoma with *MYC*  rearrangement | 0 | 0 | 2/60 (3%) |
| DLBCL | 57/70 (81%) | 112/152 (74%) | 45/60 (75%) |
| Other B-cell, CD20 positive | 6/70 (9%) | 8/152 (5%) | 6/60 (10%) |
| Other B-cell, CD20 negative | 2/70 (3%) | 3/152 (2%) | 1/60 (2%) |
| Multicentric Castleman’s disease | 0 | 1/152 (1%) | 0 |
| **EBV-association** | | | |
| EBV-associated | 29/66 (44%) | 67/144 (47%) | 23/60 (38%) |
| Non-EBV-associated | 37/66 (56%) | 77/144 (53%) | 37/60 (62%) |
| **Ann Arbor stage*** | | | |
| I | 9/70 (13%) | 30/151 (20%) | 7/60 (12%) |
| II | 9/70 (13%) | 20/151 (13%) | 9/60 (15%) |
| III | 10/70 (14%) | 22/151 (15%) | 6/60 (10%) |
| IV | 42/70 (60%) | 79/151 (52%) | 38/60 (63%) |
| **LDH** | | | |
| Elevated | 51/68 (75%) | 97/150 (65%) | 34/60 (57%) |
| **Nodal disease** | 40/57 (70%) | 110/151 (73%) | 46/60 (76%) |
| **Extranodal disease** | 54/67 (81%) | 108/151 (72%) | 56/60 (93%) |
| Gastrointestinal | 17/64 (27%) | 43/151 (28%) | 28/60 (47%) |
| Liver | 12/64 (19%) | 34/151 (23%) | 16/60 (27%) |
| Lung | 10/64 (16%) | 26/151 (17%) | 11/60 (18%) |
| Kidney | 3/64 (5%) | 4/151 (3%) | 3/60 (5%) |
| Bone marrow | 7/64 (11%) | 12/151 (8%) | 6/60 (10%) |
| Graft | 10/64 (16%) | 13/151 (9%) | 10/60 (17%) |
| **International Prognostic Index (IPI)** | | | |
| <3 | 36/65 (55%) | 88/143 (62%) | 37/60 (62%) |
| ≥3 | 29/65 (45%) | 55/143 (38%) | 23/60 (38%) |
| **ECOG performance status^†^** | | | |
| 0 | 31/68 (46%) | 40/144 (28%) | 22/60 (37%) |
| 1 | 18/68 (26%) | 66/144 (46%) | 25/60 (42%) |
| 2 | 13/68 (19%) | 32/144 (22%) | 13/60 (22%) |
| 3 | 5/68 (7%) | 6/144 (4%) | 0 |
| 4 | 1/68 (1%) | 0 | 0 |

DLBCL: diffuse large B-cell lymphoma, EBV: Epstein-Barr Virus, ECOG: Eastern Cooperative Oncology Group.

**Supplementary Table 2:** Baseline characteristics: Patients treated in the low-risk-group of the PTLD-2 trial (n=21) and the pre-specified comparator group for EFS (n=25), patients in CR (or PR with <3 initial IPI risk factors) after rituximab IV induction who were treated with CHOP consolidation in the PTLD-1 sequential treatment trial.(Trappe et al., 2012)

|  | **PTLD-1 sequential treatment “low-risk”(n=25)** | **PTLD-2**  **low-risk**  **(n=21)** | **p** |
| --- | --- | --- | --- |
| **Response to rituximab induction** | | | |
| CR | 14/25 (56%) | 5/21 (24%) | 0.027 |
| PR | 11/25 (44%) | 16/21 (76%) |  |
| **Age/years: median (range)** | 47.7 (16–70) | 43.3 (18–79) | 0.851 |
| ≥ 60 years | 4/25 (16%) | 5/21 (24%) | 0.506 |
| **Male** | 18/25 (72%) | 15/21 (71%) | 0.966 |
| **Transplant type** | | | |
| Kidney | 9/25 (36%) | 13/21 (62%) | 0.035 |
| Liver | 4/25 (16%) | 1/21 (5%) |  |
| Heart | 10/25 (40%) | 1/21 (5%) |  |
| Lung | 1/25 (4%) | 3/21 (14%) |  |
| Kidney + pancreas | 1/25 (4%) | 2/21 (10%) |  |
| Liver + lung | 0/25 | 1/21 (5%) |  |
| **Time from transplantation to PTLD/years: median (range)** | 7.6 (0.3 – 13.4) | 9.1 (0.3–31.2) | 0.501 |
| < 1 year | 4/25 (16%) | 6/21 (29%) | 0.303 |
| **Histology** | | | |
| Polymorphic | 0/25 | 2/21 (10%) |  |
| Monomorphic | 25/25 (100%) | 19/21 (90%) |  |
| Burkitt | 1/25 (4%) | 0/21 |  |
| DLBCL | 18/25 (72%) | 16/21 (76%) |  |
| Other B-cell, CD20 positive | 6/25 (24%) | 3/21 (14%) |  |
| **EBV-associated** | 11/25 (44%) | 11/21 (52%) | 0.571 |
| **Ann Arbor stage** | | | |
| I | 6/25 (24%) | 4/21 (19%) | 0.753 |
| II | 6/25 (24%) | 5/21 (24%) |  |
| III | 3/25 (12%) | 1/21 (5%) |  |
| IV | 10/25 (40%) | 11/21 (52%) |  |
| **LDH** | | | |
| Elevated | 14/25 (56%) | 6/21 (29%) | 0.062 |
| **Nodal disease** | 13/21* (62%) | 14/21 (67%) | 0.747 |
| **Extranodal disease** | 19/24* (79%) | 21/21 (100%) | 0.027 |
| Gastrointestinal | 4/23* (17%) | 8/21 (38%) | 0.124 |
| Liver | 2/23* (9%) | 3/21 (14%) | 0.560 |
| Lung | 1/23* (4%) | 4/21 (19%) | 0.125 |
| Kidney | 0/23* | 2/21 (10%) | 0.130 |
| Bone marrow | 2/23* (9%) | 2/21 (10%) | 0.924 |
| Graft | 0/23* | 4/21 (19%) | 0.028 |
| **International Prognostic Index (IPI)** | | | |
| ≥3 | 3/24* (13%) | 2/21 (10%) | 0.751 |
| **ECOG performance status** | | | |
| 0 | 17/25 (68%) | 9/21 (43%) | 0.208 |
| 1 | 6/25 (24%) | 10/21 (48%) |  |
| 2 | 2/25 (8%) | 2/21 (10%) |  |

DLBCL: diffuse large B-cell lymphoma, EBV: Epstein-Barr Virus, ECOG: Eastern Cooperative Oncology Group. *data not available in the missing patients

**Supplementary Table 3:** Baseline characteristics: Patients treated with rituximab monotherapy consolidation in the respective low-risk groups of the PTLD-1 RSST (Trappe et al., 2017) and PTLD-2 trials. Included are 42 patients in CR after rituximab induction in the PTLD-1 RSST (37 patients) and the PTLD-2 trials (5 patients) as well as 16 patients in PR after rituximab induction with <3 IPI risk factors in the PTLD-2 trial.

|  | **Rituximab monotherapy consolidation (n=58)** | **Rituximab monotherapy consolidation after CR (n=42)** | **Rituximab monotherapy consolidation after PR and <3 IPI risk factors (n=16)** | **p (CR vs. PR)** |
| --- | --- | --- | --- | --- |
| **Response to rituximab induction** | | | | |
| CR | 42/58 (72%) | 42 | 0 |  |
| PR | 16/58 (28%) | 0 | 16 |  |
| **Age/years: median (range)** | 48.1 (18–79) | 48.7 (20–78) | 37.6 (18–79) | 0.175 |
| ≥ 60 years | 15/58 (26%) | 12/42 (29%) | 3/16 (19%) | 0.445 |
| **Male** | 44/58 (76%) | 34/42 (81%) | 10/16 (63%) | 0.142 |
| **Transplant type** | | | | |
| Kidney | 34/58 (59%) | 23/42 (55%) | 11/16 (69%) | 0.312 |
| Liver | 7/58 (12%) | 7/42 (17%) | 0/16 |  |
| Heart | 3/58 (5%) | 3/42 (7%) | 0/16 |  |
| Lung | 9/58 (16%) | 6/42 (14%) | 3/16 (19%) |  |
| Kidney + pancreas | 2/58 (3%) | 1/42 (2%) | 1/16 (6%) |  |
| Liver + lung | 1/58 (2%) | 0/42 | 1/16 (6%) |  |
| Heart + kidney | 1/58 (2%) | 1/42 (2%) | 0/16 |  |
| Heart + lung | 1/58 (2%) | 1/42 (2%) | 0/16 |  |
| **Time from transplantation to PTLD/years: median (range)** | 3.8 (0.2 – 31.2) | 2.1 (0.2 – 27.9) | 7.8 (0.3–31.2) | 0.334 |
| < 1 year | 22/58 (38%) | 17/42 (41%) | 5/16 (31%) | 0.517 |
| **Histology** | | | | |
| Polymorphic | 8/58 (14%) | 6/42 (14%) | 2/16 (13%) |  |
| Monomorphic | 50/58 (86%) | 36/42 (86%) | 14/16 (88%) |  |
| Burkitt | 1/58 (2%) | 1/42 (2%) | 0/16 |  |
| DLBCL | 43/58 (74%) | 31/42 (74%) | 12/16 (75%) |  |
| Other B-cell, CD20 positive | 6/58 (10%) | 4/42 (10%) | 2/16 (13%) |  |
| **EBV-associated** | 34/57* (60%) | 24/41* (59%) | 10/16 (63%) | 0.784 |
| **Ann Arbor stage** | | | | |
| I | 15/58 (26%) | 12/42 (29%) | 3/16 (19%) | 0.251 |
| II | 10/58 (17%) | 5/42 (12%) | 5/16 (31%) |  |
| III | 3/58 (5%) | 3/42 (7%) | 0/16 |  |
| IV | 30/58 (52%) | 22/42 (52%) | 8/16 (50%) |  |
| **LDH** | | | | |
| Elevated | 30/58 (52%) | 26/42 (62%) | 4/16 (25%) | 0.012 |
| **Nodal disease** | 37/58 (64%) | 27/42 (64%) | 10/16 (63%) | 0.899 |
| **Extranodal disease** | 47/58 (81%) | 31/42 (74%) | 16/16 (100%) | 0.023 |
| Gastrointestinal | 16/58 (28%) | 12/42 (29%) | 4/16 (25%) | 0.786 |
| Liver | 13/58 (22%) | 10/42 (24%) | 3/16 (19%) | 0.680 |
| Lung | 11/58 (19%) | 7/42 (17%) | 4/16 (25%) | 0.469 |
| Kidney | 3/58 (5%) | 1/42 (2%) | 2/16 (13%) | 0.120 |
| Bone marrow | 4/58 (7%) | 3/42 (7%) | 1/16 (6%) | 0.905 |
| Graft | 7/58 (12%) | 3/42 (7%) | 4/16 (25%) | 0.062 |
| **International Prognostic Index (IPI)** | | | | |
| ≥3 | 13/58 (22%) | 13/42 (31%) | 0/16 | 0.012 |
| **ECOG performance status** | | | | |
| 0 | 26/55* (47%) | 19/39 (49%) | 7/16 (44%) | 0.219 |
| 1 | 22/55* (40%) | 13/39 (33%) | 9/16 (56%) |  |
| 2 | 6/55* (11%) | 6/39 (15%) | 0/16 |  |
| 3 | 1/55* (2%) | 1/39 (3%) | 0/16 |  |

DLBCL: diffuse large B-cell lymphoma, EBV: Epstein-Barr Virus, ECOG: Eastern Cooperative Oncology Group. *data not available in the missing patient(s)

**Supplementary Table 4:** Multivariate Cox-regression analyses for OS and TTP. Factors included in these analyses were: Thoracic organ transplant (heart, lung or combination involving heart or lung), overall response to rituximab (CR/PR vs. SD/PD) and baseline IPI ≥3.

| **OS** | | | | |
| --- | --- | --- | --- | --- |
| **Cox regression model n=58, Step 1, p<0.001** | **Risk factor** | **p** | **HR** | **95% CI** |
|  | IPI ≥3 | 0.005 | 4.640 | 1.578–13.644 |
|  | Thoracic organ transplant | 0.012 | 3.448 | 1.317–9.026 |
|  | Response to rituximab | 0.096 | 0.415 | 0.147–1.170 |
|  |  |  |  |  |
| **TTP** | | | | |
| **Cox regression model n=58, Step 3, p=0.016** | **Risk factor** | **p** | **HR** | **95% CI** |
|  | IPI ≥3 | 0.026 | 4.333 | 1.188–15.807 |

OS indicates overall survival, TTP time to progression, HR hazard ratio, and CI confidence interval. Thoracic organ transplant (HR=0.771, p=0.699, step 2) and response to rituximab (HR=0.867, p=0.811, step 1) were excluded from the TTP model.

**Supplementary Table 5:** Adverse events with a frequency > 2% in the at-risk population of the PTLD-2 trial (n=59). If one patient had repeated multiple identical AEs of different grading, only the highest graded AE was included.

| **Adverse event**  (affected patients/patients at risk) | all grades | grade 1 | grade 2 | grade 3 | grade ≥4 |
| --- | --- | --- | --- | --- | --- |
| *Adverse events of special interest* | | | | | |
| Infection | 34/59 (58%) | 4/59 (7%) | 5/59 (8%) | 16/59 (27%) | 9/59 (15%) |
| Infusion related reactions to rituximab IV | 8/59 (14%) | 4/59 (7%) | 4/59 (7%) | 0/59 (0%) | 0/59 (0%) |
| Local skin reaction at injection site (rituximab SC) | 4/59 (7%) | 4/59 (7%) | 0/59 (0%) | 0/59 (0%) | 0/59 (0%) |
| Progressive multifocal Leukencephalopathy | 0/59 (0%) |  |  |  |  |
| Pneumocystis jirovecii pneumonia | 0/59 (0%) |  |  |  |  |
| Skin reactions such as toxic epidermal necrolysis and Stevens-Johnson syndrome | 0/59 (0%) |  |  |  |  |
|  | | | | | |
| Peripheral neuropathy | 14/59 (24%) | 9/59 (15%) | 5/59 (8%) | 0/59 (0%) | 0/59 (0%) |
| Tumour lysis syndrome | 2/59 (3%) | 0/59 (0%) | 0/59 (0%) | 2/59 (3%) | 0/59 (0%) |
| Multi-organ failure | 2/59 (3%) | 0/59 (0%) | 0/59 (0%) | 0/59 (0%) | 2/59 (3%) |
| *Haematotoxicity* | | | | | |
| Leukopenia | 29/59 (49%) | 3/59 (5%) | 4/59 (7%) | 6/59 (10%) | 16/59 (27%) |
| Anaemia | 32/59 (54%) | 5/59 (8%) | 13/59 (22%) | 11/59 (19%) | 3/59 (5%) |
| Thrombocytopenia | 31/59 (53%) | 14/59 (24%) | 4/59 (7%) | 10/59 (17%) | 3/59 (5%) |
| *Renal toxicity* | | | | | |
| Acute renal failure | 17/59 (29%) | 5/59 (8%) | 3/59 (5%) | 6/59 (10%) | 3/59 (5%) |
| Hyperuricemia | 3/59 (5%) | 3/59 (5%) | 0/59 (0%) | 0/59 (0%) | 0/59 (0%) |
| Hyponatraemia | 3/59 (5%) | 1/59 (2%) | 1/59 (2%) | 1/59 (2%) | 0/59 (0%) |
| Hyperkalaemia | 2/59 (3%) | 2/59 (3%) | 0/59 (0%) | 0/59 (0%) | 0/59 (0%) |
| *Gastrointestinal toxicity* | | | | | |
| Mucositis | 4/59 (7%) | 0/59 (0%) | 3/59 (5%) | 1/59 (2%) | 0/59 (0%) |
| Elevation of liver enzymes | 6/59 (10%) | 1/59 (2%) | 4/59 (7%) | 1/59 (2%) | 0/59 (0%) |
| Abdominal pain | 4/59 (7%) | 4/59 (7%) | 0/59 (0%) | 0/59 (0%) | 0/59 (0%) |
| Nausea | 6/59 (10%) | 1/59 (2%) | 5/59 (8%) | 0/59 (0%) | 0/59 (0%) |
| Emesis | 5/59 (8%) | 1/59 (2%) | 2/59 (3%) | 2/59 (3%) | 0/59 (0%) |
| Constipation | 6/59 (10%) | 1/59 (2%) | 2/59 (3%) | 3/59 (5%) | 0/59 (0%) |
| Ileus | 3/59 (5%) | 0/59 (0%) | 2/59 (3%) | 0/59 (0%) | 1/59 (2%) |
| Diarrhoea | 15/59 (25%) | 6/59 (10%) | 6/59 (10%) | 3/59 (5%) | 0/59 (0%) |
| Gastrointestinal haemorrhage | 7/59 (12%) | 2/59 (3%) | 1/59 (2%) | 0/59 (0%) | 4/59 (7%) |
| Gastrointestinal perforation | 2/59 (3%) | 0/59 (0%) | 0/59 (0%) | 2/59 (3%) | 0/59 (0%) |
| *All other toxicities by frequency* | | | | | |
| Drug fever | 7/59 (12%) | 3/59 (5%) | 3/59 (5%) | 1/59 (2%) | 0/59 (0%) |
| Arthralgia | 4/59 (7%) | 1/59 (2%) | 1/59 (2%) | 2/59 (3%) | 0/59 (0%) |
| Back pain | 4/59 (7%) | 2/59 (3%) | 0/59 (0%) | 1/59 (2%) | 1/59 (2%) |
| Fatigue | 4/59 (7%) | 4/59 (7%) | 0/59 (0%) | 0/59 (0%) | 0/59 (0%) |
| Rash | 4/59 (7%) | 3/59 (5%) | 1/59 (2%) | 0/59 (0%) | 0/59 (0%) |
| Headache | 3/59 (5%) | 1/59 (2%) | 1/59 (2%) | 1/59 (2%) | 0/59 (0%) |
| Lung infiltrates | 3/59 (5%) | 0/59 (0%) | 2/59 (3%) | 1/59 (2%) | 0/59 (0%) |
| Hyperglycaemia | 3/59 (5%) | 1/59 (2%) | 2/59 (3%) | 0/59 (0%) | 0/59 (0%) |
| Epistaxis | 2/59 (3%) | 2/59 (3%) | 0/59 (0%) | 0/59 (0%) | 0/59 (0%) |
| Dizziness | 2/59 (3%) | 2/59 (3%) | 0/59 (0%) | 0/59 (0%) | 0/59 (0%) |
| Deep vein thrombosis | 2/59 (3%) | 0/59 (0%) | 2/59 (3%) | 0/59 (0%) | 0/59 (0%) |
| Hypertension | 2/59 (3%) | 1/59 (2%) | 1/59 (2%) | 0/59 (0%) | 0/59 (0%) |

**Supplementary Figure S1: Time-to event outcomes in the intention-to treat population of the PTLD-2 trial (n=60, solid line, median follow-up 2.8 years) in comparison to the ITT populations treated in the PTLD-1 trial with sequential therapy (n=70, broken line, median follow-up 5.1 years, Trappe et al., 2012) and risk-stratified sequential therapy (n=152, dashed line, median follow-up 4.8 years, Trappe et al., 2017).** Numbers at risk are indicated at the bottom of each graph. A: Response duration (patients in CR or PR, n=227, p=0.494). B: Time to progression (all patients, p=0.551). C: Overall survival (all patients, p=0.574). D: Progression-free survival (all patients, p=0.397).

**Supplementary Figure S2**: **Time-to event outcomes in the intention-to treat population of the PTLD-2 trial by allocated risk group (n=58).** Median time of follow-up was ≥ 2.8 years for each risk group. Low-risk group (n=21, solid line); high-risk group (n=28, broken line), very-high-risk group (n=9, dashed line). Numbers at risk per group are indicated at the bottom of each graph. A: Response duration (patients in CR or PR, n=45, p=0.905). B: Time to progression (n=58, p=0.136). C: Overall survival (n=58, p<0.001). D: Progression-free survival (n=58, p<0.001).

**Supplementary Figure S3:** Time to progression and overall survival by EBV-association of PTLD. EBV-positive PTLD (n=23, solid line); EBV-negative PTLD (n=37, dashed line). Numbers at risk for both populations (EBV-positive and EBV-negative) are indicated at the bottom of each graph. A: Time to progression (p=0.952). B: Overall survival (p=0.867).

**Supplementary Figure S4:** Time to progression and overall survival by patient sex. Female patients (n=16, solid line); Male patients (n=44, dashed line). Numbers at risk for both populations are indicated at the bottom of each graph. A: Time to progression (p=0.772). B: Overall survival (p=0.668).

**Supplementary Figure S5:** Time to progression and overall survival in the PTLD-2 trial by number of international prognostic index risk factors < 3 (n=37, broken line) and ≥ 3 (n=23, solid line). All 60 patients of the intention-to-treat population are included. Numbers at risk for both populations are indicated at the bottom of each graph.

A: Time to progression (p=0.016). B: Overall survival (p=0.001).
